# Editorial: Advances in the Integration of Brain-Machine Interfaces and Robotic Devices

**DOI:** 10.3389/frobt.2021.653615

**Published:** 2021-03-10

**Authors:** Luca Tonin, Emanuele Menegatti, Damien Coyle

**Affiliations:** ^1^ Department of Information Engineering, University of Padua, Padua, Italy; ^2^ Intelligent Systems Research Centre, School of Computing, Engineering and Intelligent Systems, Ulster University, Derry, United Kingdom

**Keywords:** Brain-machine interface, robotics, shared-control, human-robot interaction, assistive devices

Recent advances in noninvasive Brain-Machine Interfaces (BMIs) have demonstrated the potential impact of directly interfacing the brain with machines. The ultimate translational goal of BMI systems is to enable people suffering from severe motor disabilities to control a new generation of neuroprostheses and, thus, (re)gain their own independence.

Many studies have already demonstrated the feasibility of the BMI technology with different kinds of assistive devices, designed to restore communication (e.g., virtual keyboard) or to enable the control of robotic applications (e.g., wheelchairs, telepresence robots, robotic arms, and drones). However, despite great progress, the integration of the BMI and robotics is still in its infancy and translational impact is low.

The BMI community has predominantly focused on exploring novel algorithms to decode the user’s intentions from neural patterns with a focus on enhancing the robustness and the reliability of the BMI system. However, the process of how the estimated intentions of the user are translated by the intelligent robotic device into real and daily-based situations is often neglected. This largely affects the translational impact of the BMI technology. The latest advances in the field of robotics may help address this challenge by exploiting novel human–robot interaction theories and by providing insights and solutions from a new and different perspective.

This special topic sought original contributions that explicitly take into account the cross-cutting aspects in BMI and robotics research including but not limited to BMI control of navigation robots, BMI control of robotic prosthetic limbs, BMI-driven assistive technology for end users, translational aspects in BMI-controlled devices, shared-control strategies for the BMI, contextualized robotic behaviors, long-term human–robot interaction (BMI–robot interaction), semi-autonomous robot behaviors, evaluation of BMI-driven robotics in real-world scenarios, and real-time detection of possible targets in real-world scenarios. All typologies of closed-loop BMI systems (e.g., based on exogenous stimulation or self-paced paradigms) were solicited if they focused on the integration of BMI and robotics devices. We are pleased with the interest in the topic and the collection of studies presented, which include two state-of-the-art reviews, one on neural driven rehabilitation robotics for lower limb gait rehabilitation and another human affective states when interfacing with robotic devices, as well as five novel studies investigating a range of BMI robotic learning scenarios and signal decoding in invasive and noninvasive BMIs, all of which highlight opportunities and challenges to advance the integration of Brain-Machine Interfaces and Robotic devices.

In their review, Alirmardani and Hiraki focused on the current employment of BMIs in human–robot interaction applications. They illustrated the state of the art of passive BMIs and the current challenges to monitor and decode cognitive load, attention level, perceived errors, and emotional states in real time.

Kim et al. investigated the influence and the effect of human supervision on robot learning during pick-and-place tasks. In the proposed experimental scenario, two human–robot interfaces were provided: the first one based on human gestures to decode the human’s intent and the second based on error-related potentials to provide the human’s intrinsic feedback of the performed robot action. They demonstrated that such a human–robot interaction promoted robotic learning and the concurrent online adaptation, especially when prior knowledge about the task was provided.

Monitoring robot behavior through the evaluation of possible mistakes may be not the only way to foster the learning process of intelligent devices. Wirth et al. showed the possibility of exploiting a single-trial P300-based BMI to discriminate when a virtual robot has reached a predefined destination during navigation tasks. They proposed this approach as part of a learning-based system to enhance the efficacy and efficiency of BMI-driven applications for navigation.

Similarly, Kolkhorst et al. showed that a robotic agent could improve the usability of an event-related potential BMI by obviating the traditional need of an external screen for stimulus presentation. They exploited a robotic arm to present stimuli by highlighting objects in a realistic environment with a laser pointer. The proposed classification method, based on specialized classifiers in the Riemann tangent space, reported not only high accuracy but also robustness to both heterogeneous and homogeneous objects.

Beyond exploiting the “human-in-the-loop” approach to monitor robot behaviors, BMI systems can be also used to directly control the movement of robotic actuators. While most studies to date demonstrated the feasibility of mentally driving a single assistive device, Huang et al. proposed a hybrid BMI system to control an integrated wheelchair-robotic arm system. A motor imagery BMI was used to deliver navigation commands to the wheelchair and an electrooculogram-based interface for the control of the robotic arm. Interestingly, the system allows the users to voluntarily renew the classification parameters during online operations by means of a specific sequence of commands.

In Kim et al., authors proposed a new decoding algorithm based on deep canonical correlation analysis and neuronal firing rate activities that improves the kinematic reconstruction in a 2D arm reaching task performed by nonhuman primates. The algorithm was designed to identify the best kinematics-related canonical variables of neuronal activity via deep learning–based approaches. As highlighted in the study, the prediction of kinematic parameters of a prosthetic device from neural activities can have profound consequences in BMI clinical applications.

Finally, Lennon et al. conducted a systematic review of the current state of the art and limitations of neural driven robotic gait devices in stroke rehabilitation. Despite identifying a limited number of promising studies to date, the review highlighted wide heterogeneity in the reporting and the purpose of neurobiosignal utilization during robotic gait training after a stroke and the lack of standardized protocols. A quick reference guide (the DESIRED Checklist) is proposed to identify a minimum reporting data set as a standard for future studies in order to maximize the translational impact of the technology.

In summary, in the BMI field, the role of the robotic intelligence is often underestimated by relegating the robotic device to a mere actuator of the user’s commands. This collection highlighted challenges to be addressed and potential solutions, standards to adhere to when undertaking studies, and the importance of further investigating the potential bidirectional human–robot interactions in BMI applications in order to improve the overall efficiency of these novel interfaces and to design a new generation of neuroprosthetic devices.

